# Unused, expired pharmaceuticals and their disposal practices among the general public in Burdur-Türkiye: a cross-sectional study

**DOI:** 10.1186/s12889-024-18788-0

**Published:** 2024-05-13

**Authors:** Serkan Köksoy

**Affiliations:** Mehmet Akif Ersoy University, Health Science Faculty, Burdur, Türkiye

**Keywords:** Pharmaceutical, Public health, Self medication, Türkiye

## Abstract

**Background:**

Unused pharmaceuticals are currently a public health problem. This study aimed to identify unused pharmaceuticals, research practices about the disposal methods, classify the medicines according to Anatomical Therapeutic Chemical codes (ATC) and, to determine the number of unused medicines.

**Methods:**

The study was designed as a cross-sectional study. Data were collected between April and August 2023 in Burdur-Türkiye by non-probability sampling technique (convenience method). Pharmaceuticals were classified according to ATC. Statistical Package for Social Science SPSS (V.24) package program was used for data analysis.

**Results:**

A total of 1120 people, 1005 in the first sample group and 115 in the second sample group, participated in the study. Findings of first sample group: A total of 4097 boxes of unused pharmaceuticals (4.7 ± 4.3 boxes/per capita) were detected. It was found that pharmaceuticals were stored in areas such as kitchens (59.1%) and refrigerators (38.6%), the reason for keeping them was reuse (41%), and the disposal practice was household garbage (81%). *Paracetamol* (648 boxes), *Other cold preparation* (303 boxes), *Dexketoprofen* (239 boxes), *Diclofenac* (218 boxes), *Amoxicillin and beta-lactamase inhibitor (*190 boxes) were found to be the most frequently unused pharmaceuticals. Using the unused medicines at home without consulting a physician was 94.1% (self-medication). Findings of second sample group: Of the 6189 dosage forms in 265 boxes pharmaceutical, 3132(50.6%) dosage forms were used and 3057(49.4%) were found to be unused.

**Conclusion:**

There is a significant amount and number of unused medicines in households, and self-medication is common. Medicines are not properly disposed of and some of them expire. Public information is needed. A “drug take-back system” for unused medicines can be useful in solving this problem.

## Introduction

Drugs are agents used in the treatment of diseases and are prescribed by physicians based on the patient’s diagnosis. For home treatment, medications prescribed by physicians are obtained from the pharmacy and used for treatment. However, patients may prematurely discontinue home drug treatment for various reasons. The main reasons for early discontinuation include the early recovery from disease, a change in diagnosis, a decrease in or loss of faith in the treatment, side effects of the medication, and the patient non-compliance with treatment, among others [1]. When the patients discontinue drug treatment for any reason, they may contribute to the accumulation of unused medicines in the household. The unused pharmaceuticals that accumulate in the household can be reused at different times to alleviate the symptoms of various diseases, can be given to another person, or kept on hand until the expiration date [2]. If there is lack of knowledge about the medicines that accumulate in the household, the expiration dates of the medicines may expire, which can lead to health problems when these medicines are used [3].

Several epidemiological studies have been conducted on unused pharmaceuticals. In a study conducted in Saudi Arabia, it was reported that about half of the participants accumulated unused pharmaceuticals in their households, the main reason for accumulation was to cure illness, they disposed of the pharmaceuticals by throwing them in the garbage, and a significant number of participants were uneducated about pharmaceutical disposal practices [4]. In a study conducted by Woldeyohanins et al. (2021), it was reported that more than half of the participants had unused pharmaceuticals in their homes, about 59.2% checked the expiration date, and the household trash can was used as a method of pharmaceutical disposal practices [5]. In a similar study, people were reported to have unused medicines at homes, to store them because they might need them later, and to dispose of them inappropriately [6].

While this is the case in the general public, similar findings have been reported among student in health professions (medicine, nursing, pharmacy, etc.). For example, a previous study reported that 95.7% of pharmacy students had unused medications, some of which were expired and not properly disposed [7, 8]. Improper disposal of pharmaceuticals posed an environmental hazard. The presence of pharmaceutical waste in wastewater treatment plants was indicative of this problem. A study reported that pharmaceutical metabolites and active ingredients were detected in drinking water facilities, even if at low levels [9].

There are numerous studies in the literature on unused medicines. These studies summarize data from different countries, cultures, geographies, and levels of development. In generel, these studies categorize the unused medicines by mechanism of action (antibiotics, analgesics, etc.) rather than by Anatomical Therapeutic Chemical classification (ATC). In addition, it has been observed that the number of studies reporting the amount of used and unused medicines is extremely limited. This gap in the literature needs to be addressed. Therefore, this study aimed to identify unused medications, classify them according to the ATC, research participants’ practices about these medications, and determine how much of medications such as tablets, capsules and dragees with a given quantity are used and how much is unused.

## Methods

### Study design

The study was designed as a cross-sectional study. Data were collected between April 1 and August 31, 2023 in Burdur-Türkiye. This study consists of two sample groups, the first sample group and the second sample group. The data of both groups were collected by a non-probability sampling technique (convenience method). While the online method (Google form) was used to collect the data in the first sample, the face-to-face method was used in the second sample. For first sample size, proportionally based sampling with the double design effect method was used. For second sample size, certain percentage of the calculated number of participants in the first sample group was taken as a basis. The purpose of the second sample was to determine the quantity of unused pharmaceuticals, to strengthen the findings and to enhance the literature. For some reasons, the data collected from the first sample were not collected from the second sample (Private life/privacy, protection of personal data, difficulty of voluntary participation, similar personal characteristics.etc.).

### Participant criteria

Participant criteria were determined to participate in the study. These were determined as not having any chronic disease at the time of data collection and not taking medication for any disease, having appropriate equipment for online data (computer, smartphone, tablet), living at home due to the question types, being literate (for face-to-face data collection), not being given partial or incomplete information, being above the age of 18 years and giving consent. Participant exclusion criteria were defined as not having the above-mentioned conditions and not having unused medicines.

### First sample size

A formula (*n = Z*^*2*^ x *p(1-p)/d*^*2*^ x *DEFF* (*n: sample size, Z:confidence interval, p:expected proportion, d:margin of error and DEFF: design effect)*) was used to calculate this sample size [10]. According to this formula (*1.96*^*2*^* × 0.5(1–0.5)/0.05*^*2*^* × 2n=754 × 0.1*), it was needed that at least 830 people participate in the study. Sociodemographic data, practices on unused medicines, disposal methods, number of unused medicines and generic names of medicines were collected from these sample group.

### Second sample size

For the quantity analysis, 10% (*n* = 83) of the first sample size (*n* = 830) was taken as a basis [11]. Only the generic name of pharmaceutical and quantity of unused drugs (dosage forms) were collected from this sample.

As a result, it was calculated that at least 913 people participated in the study.

### Data collection tool

Since the research consisted of two separate sample groups, two separate forms were used. The data collection form collected from the first sample included the sociodemographic characteristics of the participants, their practices about unused drugs, disposal method, generic names of drugs and the number of unused drugs. In the second sample group data collection form, only data on the quantity of unused medicines such as tablets, dragees and capsules were collected.

Description of unused drug: It is called the leftover part of the drug after treatment. This description was sent as a video to the group for online data collection. It was explained verbally to the face-to-face data collection group.

### The data collection form of the first sample group

In order to obtain data from the first sample group of the study, a questionnaire was developed based on the questions of previous studies [11–14]. The content of this form includes socio-demographics, practices about on unused medicines, disposal practices, number of unused medicines and generic names of medicines. The data collected through this form were collected online by using a Google form. The Google form was turned into a link. This link and the explanation video of the research (purpose, participant criteria, data filling examples, etc.) were sent to people as text messages via WhatsApp. In particular, groups with a large number of people registered (groups such as school, work, etc.) were targeted.

### The data collection form of the second sample group

This was a data collection form in which quantity of unused medicines in the medicines box (tablets, dragees, capsules, etc.) was recorded. The number of dosage forms of these medicines was obtained by simple subtraction (Number of dosage forms of unused medicines = number of dosage forms of medicines - number of dosage forms of medicines used). Data used for quantity analysis were collected face-to-face by using a written data collection form.

### Classification of Pharmaceuticals

Pharmaceuticals were classified according to the ATC/DDD Index 2023. This index is a very useful resource published by WHO and WHO Collaborating Centre for Drug Statistics Methodology and includes Anatomical Therapeutic Chemical (ATC) classification, Defined Daily Dose (DDD) values, codes [15].

### Data Analysis

SPSS (V.24) package program was used for data analysis. Descriptive parameters were summarized as (n) and percentage (%) where relevant [n(%)]. Mean (X̅) and standard deviation (S) were summarized as X̅±S. The relationship between the number of pharmaceuticals in the households and sociodemographic variables was tested with non-parametric tests (Mann Whitney U, Kruskal Wallis, Tamhanes T2). Statistical significance level was taken as *p* < 0.05.

## Results

A total of 1172 people participated in the study, 1057 in the first sample group (52 people were excluded) and 115 in the second sample group. The study was completed with 1120 participants.

### The findings of the first sample group (*n* = 1005)

Some people data were not included in the study due to exclusion criteria (*n* = 24, 2.3%). The number of people with unused pharmaceuticals was found to be 28(2.6%) (97.4% of participants have unused medicines). Therefore, study was completed with 1005 participants (Age_mean_ 36.9 ± 12.3 years). A total of 4097 boxes of unused pharmaceuticals were observed (4.7 ± 4.3 boxes/per capita).

When evaluating the number of unused pharmaceuticals and the sociodemographic characteristics of the participants (*n* = 1005), no statistical difference was observed in gender variable (*p* = 0.089). However, statistical differences were observed in marital status (lower in singles), place of residence (lower in rural areas), and economic status (Income < Expense) (*p* < 0.001, *p* < 0.001, *p* < 0.001, respectively) as shown in Table 1.

**Table 1:** The relationship between sociodemographic variables and the number of unused pharmaceuticals (*n* = 1005)

Variable		*n*	%	Med	Mean Rank	Test	*p*
Gender	Female	603	60	3	489.38	U:113,105,500	0.089
	Male	402	40	4	520.94		
Marital Status	Single	328	32.6	3	435.89	U:88,909,500	< 0.001
	Married	677	67.4	4	533.98		
Place	City	772	76.8	3	477.15	U:70,277,000	< 0.001
	Rural	233	23.2	5	584.58		
Economic Status	Income = Expense	511	50.9	4	535.63	K-W:15,217	< 0.001^a, a,b^
	Income > Expense	113	11.3	3	489.30		
	Income < Expense	377	37.5	3	460.16		
Education Status	Primary	246	24.5	3	520.81	K-W:39,919	< 0.001^a, b,c^
	Secondary	222	22	4	596.88		
	Higher	537	53.3	3	454.25		
Employment status	Working	579	57.5	3	488.64	U:115,102,000	0.086
	Not Working	426	42.3	3	520.17		

n: Participants, %: percentage of participants, Med: Median of the number of pharmaceuticals, U: Mann-Whitney U Test, K-W: Kruskal Wallis H Test, Posthoc: Tamhanes T2 (shoved letters and according to the dependent variable, respectively)

It was found that the options of kitchen (59.1%) as the room where pharmaceuticals were kept at home, refrigerator (38.6%) as the place where pharmaceuticals were stored, family health center (42.2%) as the health facility where most pharmaceuticals were prescribed, reuse (41%) as the reason for keeping pharmaceuticals in household, family physician (48.7%) as the specialty branch where most pharmaceuticals were prescribed, were selected more than other options. The options of checking the expiry date of pharmaceuticals (72.1%), reading the package insert (56.7%) and not buying pharmaceuticals without a prescription (58.7%) were more chosen ones by the participants. Keeping pharmaceuticals in household until the expiry date (44.6%), throwing expired pharmaceuticals in the garbage (81%), thinking that improper disposal of pharmaceuticals pollutes the environment (74.8%), and The Ministry of Health is responsible for raising awareness on medicines (41.7%) were the options that were more frequently marked by the participants. Using the unused medicines at home without consulting a physician was 94.1% (self-medication). (Table 2).

**Table 2:** Participants’ practices on pharmaceuticals (*n* = 1005)

Ouestions			*n*		%	
In which room of your household do you keep your pharmaceuticals? ^*^	Kitchen	727		59		
	Living room	148		12		
	Salon	151		12.3		
	Bedroom	157		12.8		
	Bathroom-toilet	48		3.9		
In which storage area do you store your pharmaceuticals? ^*^	Refrigerator	520		38.6		
	Kitchen cabinet	372		27.6		
	Storage and other cabinets	419		31.1		
	Wardrobe	37		2.7		
In which health facility do you think most pharmaceuticals are prescribed to you? ^*^	City-state hospital	394		27		
	Training and research hospital	244		16.7		
	Family health center	616		42.2		
	Medical faculty hospital	92		6.3		
	Private health facilities	113		7.8		
Why do you keep unused pharmaceuticals in household? ^*^	I can use the pharmaceutical again after a while	630		41		
	I may need pharmaceutical in an emergency	555		36.1		
	It can be used by friends or family	273		17.8		
	Other causes	80		5.2		
Which branch do you think prescribes the most pharmaceuticals to you? ^*^	Family medicine	600		48.7		
	Emergency service	147		11.9		
	Other specialties in the hospital	486		39.4		
Do you check the expiration date of the pharmaceuticals you take?	Yes	725		72.2		
	Sometimes	173		17.2		
	No	107		10.6		
Do you read the package leaflet of the pharmaceutical you take?	Yes	570		56.7		
	Sometimes	270		26.9		
	No	165		16.4		
Do you buy pharmaceuticals without consulting a physician?	Yes	415		41.3		
	No	590		58.7		
Which pharmaceuticals do you use without consulting a physician? ^*^	Antibiotics	119		4,5		
	Analgesics	786		29,7		
	The common cold drugs	498		18,8		
	Cough syrup	199		7,5		
	Antiacids	266		10,1		
	Muscle relaxant	289		10,9		
	Vitamin	332		12,6		
	I do not use without consulting a physician	157		5,9		
What do you do with unused pharmaceuticals? ^*^	I throw it in the garbage	358		29.5		
	I give to health organizations	94		7.7		
	I give it to the pharmacy	63		5.2		
	I give it to people who need it	117		9.6		
	I keep it in household until it expires	542		44.7		
	Other	40		3.3		
What do you do with expired pharmaceuticals? ^*^	I throw it in the garbage	854		81		
	I give to health organizations	55		5.2		
	I give it to the pharmacy	43		4.1		
	I give it to people who need it	21		2		
	Other	81		7.7		
Can improper disposal of unused and expired pharmaceuticals affect the environment and health?	Yes	752		74.8		
	No opinion	231		23		
	No	22		2.2		
Who is responsible for raising awareness about proper disposal of unused and expired pharmaceuticals? ^*^	Ministry of Health	745		41.7		
	Pharmaceutical companies	361		20.2		
	Pharmacy and pharmacist	391		21.9		
	Education institutions	248		13.9		
	Other	40		2.3		

*Could be more than one option, n: Participants, %: Percantege of participants

The most unused drugs by ATC class were *Lansoprozole, Folic Acid, Propranolol, Other Cicatrizants, Levothyroxine Sodium, Amoxicillin and beta lactamase inhibitor, Dexketoprofen, Paracetamol, Metronidazole, Other Cold Medicines*. In terms of number of boxes, it was “*Paracetamol, Other Cold Medicines, Dexketoprofen, Diclofenac, Amoxicillin and beta lactamase inhibitor, Pseudoephedrine combination, Lansoprazole and Acetylsalicylic acid*” (Table 3).

**Table 3:** ATC classification of unused pharmaceuticals (Number of participants:1005)

ATC Code			ATC Name	Box	%Box		
A-Digestive System and Metabolism	A02BC03	Lansoprazole		140		21.5	
	A02AD01	Ordinary salt combinations		97		14.9	
	A02BC02	Pantoprazole		83		12.8	
	A11CC05	Colecalciferol		52		8	
	A03FA01	Metoclopramide		51		7.8	
	A02BC05	Esomeprazole		34		5.2	
	A03BB01	Butylscopolamine		32		4.9	
	A11AA01	Multivitamins and trace elements		29		4.5	
	A11DB	Vitamin B1 in combination with B6 and/or B12		18		2.7	
	A06AB06	Senna glycosides		13		2	
	A12CC30	Magnesium		12		1.8	
	A03AXX1	Silicones		10		1.5	
	Others			80		12.4	
	**Class Total**			**651**		**100**	
B-Blood and Blood Forming Organs	B03BB01	Folic Acid		13		35.1	
	Others			24		64.9	
	**Class Total**			**37**		**100**	
C-Cardiovascular System	C07AA05	Propranolol		26		17.2	
	C08DB01	Diltiazem		17		11.3	
	C03BA11	Indapamide		15		9.9	
	C10AA05	Atorvastatin		13		8.6	
	C07AB02	Metoprolol		12		7.9	
	C03CA01	Furosemide		11		7.3	
	C01EB18	Ranolazine		11		7.3	
	Others			46		30.5	
	**Class Total**			**151**		**100**	
D-Dermatological	D03AX	Other cicatrizants		43		27.2	
	D11AX	Other dermatologicals		25		15.8	
	D06AX01	Fusidik Acid		14		8.9	
	D01AC20	Imidazoles/triazoles in combinations with corticosteroids		13		8.2	
	D06AX09	Mupirocin		12		7.6	
	Others			51		32.3	
	**Class Total**			**158**		**100**	
G- Genito Urinary System	Others			9		100	
	**Class Total**			**9**		**100**	
H-Systemic Hormonal Preparations	H03AA01	Levothyroxine sodium		31		72.1	
	Others			12		27.9	
	**Class Total**			**43**		**100**	
J-Anti-infective (Systemic)	J01CR02	Amoxicillin and beta-lactamase inhibitor		190		65.3	
	J01DC02	Cefuroxime		44		15.1	
	J01MA02	Ciprofloxacin		26		8.9	
	Others			31		10.7	
	**Class Total**			**291**		**100**	
L-Antineoplastic and Immunomodulating	Others			1		100	
	**Class Total**			**1**		**100**	
M-Musculoskeletal System	M01AE17	Dexketoprofen		239		24	
	M01AB05	Diclofenac		218		21.8	
	M01AE09	Flurbiprofen		101		10.1	
	M02AA10	Ketoprofen		90		9	
	M01AE02	Naproxen		64		6.4	
	M01AE01	Ibuprofen		62		6.2	
	M01AB08	Etodolac		35		3.5	
	M02AA13	Ibuprofen-Topical		34		3.4	
	M03BB53	Chlorzoxazone, combinations excl psycholeptics		30		3	
	M03BX30	Feniramidol		26		2.6	
	M02AA26	Nimesulide -Topical		19		1.9	
	M02AA15	Diclofenac-Topical		16		1.6	
	M03BX05	Thiocolchicoside		16		1.6	
	M05BB03	Alendronic acid and colecalciferol		11		1.1	
	Others			37		3.8	
	**Class Total**			**998**		**100**	
N-Nervous System	N02BE01	Paracetamol		648		71.2	
	N02BA01	Acetylsalicylic acid		139		15.3	
	N02BB02	Metamizole sodium		53		5.8	
	N02BE51	Paracetamol, combination excl psycholeptics		36		4	
	Others			34		3.7	
	**Class Total**			**910**		**100**	
P-Antiparasitic, Insecticides and Repellents	P01AB01	Metronidazole		37		100	
	**Class Total**			**37**		**100**	
R-Respiratory System	R05X	Other cold preparation		303		38	
	R01BA52	Pseudoephedrine combinations		158		19.8	
	R06AE07	Cetirizine		51		6.4	
	R05DB13	Butamirate		40		5	
	R03DC03	Montelukast combination		37		4.6	
	R06AX17	Ketotifen		26		3.3	
	R03AC02	Salbutamol		23		2.9	
	R05CB10	Combination (Acetylcysteine, Vitamin C)		23		2.9	
	R06AB05	Pheniramine		23		2.9	
	R06AX27	Desloratadine		19		2.4	
	R05CB01	Acetylcysteine		17		2.1	
	R03BA02	Budesonide		15		1.9	
	R03CC53	Terbutaline combinations		11		1.4	
	Others			52		6.4	
	**Class Total**			**798**		**100**	
S- Sensory Organs	Others			13		100	
	**Class Total**			**13**		**100**	
		**Total**		**4097**		**100**	

n = number of pharmaceuticals (box), %=percentage of the number of pharmaceuticals. Pharmaceuticals with fewer than 10 were given as “others” in the table. Pharmaceuticals in the Others section was summarized separately as ATC name, ATC code and number of pharmaceuticals (For example: Zinc sulfate(A12CB01):9). A-Others(*n* = 80):Benzidamine(A01AD02):9, Lactulose(A06AD11):9, Zinc sulfate(A12CB01):9, Nifuroxazide(A07AX03):8, Tioctic acid(A16AX01):7, Pinaverium(A03AX04):6, Sodium fluoride(A01AA01):6, Famotidine(A02BA03):5, Orlistat(A08AB01):3, Rabeprazole(A02BC04):3, Multivitamins and other minerals, incl. combinations(A11AA03):2, Otilonium bromide(A03AB06):2, Multienzymes(lipase, protease etc.)(A09AA02):2, Dexpanthenol(A11HA30):1, Domperidone(A03FA03):1, Glibenclamide(A10BB01):1, Glibornuride(A10BB04):1, Glycerol(A06AG04):1, Magnesium hydroxide(A02AA04):1, Mebeverine(A03AA04):1, Various(A01AD11):1, Pyridoxine(A11HA02):1. B-Others(*n* = 24):Cyanocobalamin(B03BA01):8, Ferrous amino acid complex and folic acid(B03AD01):7, Clopidogrel(B01AC04):3, Ticagrelor(B01AC24):1, Apixaban(B01AF02):1, Tranexamic acid(B02AA02):1, Ferrous glycine sulfate(B03AA01):1, Ferrous fumarate(B03AA02):1, Cyanocobalamin, combinations(B03BB51):1. C-Others(*n* = 46):Doxazosin(C02CA04):7, Rosuvastatin(C10AA07):6, Candesartan(C09CA06):4, Nebivolol(C07AB12):3, Amlodipine(C08CA01):2, Captopril(C09AA01):2, Lercanidipine(C08CA13):2, Digoxin(C01AA05):2, Atenolol(C07AB03):1, Benidipin(C08CA15):1, Calcium Dobesilat(C05BX01):1, Organo-heparinoid(C05BA01):1, Hydrocortisone(C05AA01):1, Quinapril and diuretics(C09BA06):1, Lisinopril(C09AA03):1, Losartan and diuretics(C09DA01):1, Olmesartan mdeoxomil and amlodipine(C09DB02):1, Pentoxifylline(C04AD03):1, Pitavastatin(C10AA08):1, Other preparations, combinations(C05AX03):1, Spironolactone(C03DA01):1, Hydrochlorothiazide combinations(C03AX01):1, Torasemide(C03CA04):1, Trimetazidine(C01EB15):1, Valsartan and diuretics(C09DA03):1, Zofenopril(C09AA15):1. D-Others(*n* = 51):Silver sulfadiazine(D06BA01):8, Centella asiatica herba(D03AX14):7, Carbamide combinations(D02AE51):5, Clobetasol(D07AD01):3, Oxytetracycline(D06AA03):3, Sertaconazole(D01AC14):3, Terbinafine(D01BA02):3, Diphenhydramine(D04AA32):2, Nadifloxacin(D10AF05):2, Wart and anti-corn preparations (D11AF):2, Acyclovir(D06BB03):1, Other antibiotics for topical use (D06AX):1, Betamethasone(D07AC01):1, Butenafine(D01AE23):1, Hydroquinone(D11AX11):1, Hydrocortisone(D07AA02):1, Isotretinoin combinations(D10AD54):1, Clobetasone(D07AB01):1, Methylprednisolone aceponate(D07AC14):1, Mometasone(D07AC13):1, Naftifine(D01AE22):1, Terbinafine(D01AE15):1, Oxiconazole(D01AC11):1. G-Others(*n* = 9):Clotrimazole(G01AF02):2, Progesterone(G03DA04):2, Dydrogesteron(G03DB01):2, Imidazole derivates and corticosteroids(G01BF):1, Estradiol(G03CA03):1, Darifenacin(G04BD10):1. H-Others(*n* = 12):Dexamethasone(H02AB02):6, Prednisolone(H02AB06):5, Betamethasone(H02AB01):1. J-Others(*n* = 31):Cefaclor(J01DC04):8, Azithromycin(J01FA10):5, Clarithromycin(J01FA09):4, Cefixime(J01DD08):3, Doxycycline(J01AA02):2, Cefprozil(J01DC10):1, Sulfamethoxazole and trimethoprim (J01EE01):1, Lincomycin(J01FF02):1, Levofloxacin(J01MA12):1, Fusidic acid(J01XC01):1, Fluconazole(J02AC01):1, Rifamycin(J04AB03):1, Aciclovir(J05AB01):1, Valaciclovir(J05AB11):1. L-Others(*n* = 1):Leflunamide(L04AA13):1 M-Others(*n* = 37):Acemetacin(M01AB11):7, Thiocolchicoside, combinations(M03BX55):7, Preparations with salicylic acid derivatives(M02AC):5, Colchicine(M04AC01):5, Phenprobamate, combinations excl. psycholeptics(M03BA51):2, Thiocolchicoside(M03BX05):2, Indomethacin(M01AB01):2, Meloxicam(M01AC06):2, Benzidamine(M02AA05):1, Etofenamate(M02AA06):1, Piroxicam(M02AA07):1, Methocarbamol, combinations excl. psycholeptics(M03BA53):1, Tizanidine(M03BX02):1. N-Others(*n* = 34):Paracetamol, combinations with psycholeptics(N02BE71):8, Sertraline(N06AB06):5, Eletriptan(N02CC06):3, Frovatriptan(N02CC07):2, Paroxetine(N06AB05):2, Methylphenidate(N06BA04):2, Flunarizine(N07CA03):2, Acetylsalicylic acid, combinations excl. psycholeptics(N02BA51):1, Ergotamine(N02CA02):1, Topiramate(N03AX11):1, Gabapentin(N0BF01):1, Lacosamide(N03AX18):1, Diazepam(N05BA01):1, Escitalopram(N06AB10):1, Trazodone(N06AX05):1, Duloxetine(N06AX21):1, Lamotrigine(N03AX09):1. R-Others(*n* = 52):Oxymetazoline(R01AA05):10, Salbutamol and sodium cromoglicate (R03AK04):7, Cyproheptadine(R06AX02):4, Oxolamine(R05DB07):3, Rupatadine(R06AX28):3, Diphenhydramine(R06AA2):2, Beclomethasone(R01AD01):2, Mometasone(R01AD09):2, Fluticasone(R03BA05):2, Theophylline(R03DA04):2, Montelukast(R03DC03):2, Ebastine(R06AX22):2, Fexofenadine(R06AX26):2, Levodropropizine(R05DB27):2, Xylometazoline(R01AA07):1, Azelastine(R01AC03):1, Pseudoephedrine(R01BA02):1, Formoterol and budesonide(R03AK07):1, Formoterol and beclometasone(R03AK08):1, Doxylamine(R06AA09):1, Bilastine(R06AX29):1. S-Others(*n* = 13):Fusidic acid(S01AA13):3, Ketotifen(S01GX08):3, Tobramycin(S01AA12):2, Artificial tears and other indifferent preparations(S01XA20):2, Gentamicin(S03AA06):2, Corticosteroids and antiinfectives in combination(S01CA01):1 (Table 3)

### The findings of the second sample group (*n* = 115)

It was found that there were 265 boxes (6189 dosage forms in the boxes) of unused pharmaceuticals (3.8 ± 5 boxes/per capita) in in households of these participants. Of the 265 boxes medicines, 74(27.9%) were found to be expired. The most *Pseudoephedrine combination* (23 boxes) and *Other cold preparation* (23 boxes) and *Dexketoprofen* (20 boxes) drugs were found to be unused drugs. The percentage of unused drugs was highest in class D(79%), P(55.4%) and N(54.6%), while used drugs were in class G(76.9%), C(69.6%) and A(53.9%) (Table 4).

Among the prescribed pharmaceuticals, pharmaceuticals in “Class G” were the most commonly used (76.9%), while pharmaceuticals in “Class D” were the least used (21%). The highest number of unused pharmaceuticals was found to be in “Class R” (929 dosage forms), followed by “Class M” drugs (682 dosage forms). Of the pharmaceuticals prescribed to the participants (6189 dosage forms), 3132(50.6%) were used and 3057(49.4%) were unused (Table 4).

**Table 4:** An analysis of unused pharmaceuticals (Number of participants = 115)

ATC Code-ATC Name	Number of pharmaceuticals	Number of Dosage Forms	Used	Unused
A12CB01 Zinc Sulfate	6	220	82(37)	138(63)
A11DB Vitamin B1 in combinations with B6 and/or B12	6	210	112(53)	98(47)
A02BC05 Esomeprazole	5	140	93(66)	47(34)
A09AA02 Multienzymes (lipase, protease etc.)	1	100	70(70)	30(30)
A02BC02 Pantoprazole	3	84	44(52)	40(48)
A02AD04 Hydrotalcite	2	80	61(76)	19(24)
A03BB01 Butylscopolamine	3	60	20(33)	40(67)
A03FA01 Metoclopramide	2	60	5(8)	55(92)
A03AX13 Silicones	1	50	38(76)	12(24)
A02AB04 Dihydroxialumini sodium carbonate	1	48	36(75)	12(25)
A03AX58 Alverine, Combinations	1	40	20(50)	20(50)
A03AB06 Otilonium Bromide	1	31	26(84)	5(16)
A16AX01 Thioctic Acid	1	30	15(50)	15(50)
A12CC30 Magnesium	1	30	22(73)	8(27)
A02BC03 Lansoprazole	1	28	10(36)	18(64)
A02BC04 Rabeprazole	1	20	1(5)	19(95)
A04AD Other antiemetics	2	32	29(91)	3(9)
A07FA02 Saccharomyces Boulardii	1	10	2(20)	8(80)
**Class Total**	**39**	**1273(100)**	**686(53.9)**	**587(46.1)**
B02AA02 Tranexamic Acid	1	50	17(34)	33(66)
B03AA02 Ferrous Fumarate	1	30	22(73)	8(27)
**Class Total**	**2**	**80(100)**	**39(48.8)**	**41(51.2)**
C09CA06 Candesartan	3	84	66(79)	18(21)
C05CA53 Diosmin, combination	1	60	50(83)	10(17)
C04AD03 Pentoxifylline	1	20	10(50)	10(50)
C05AX03 Other preparations, combinations	2	20	7(35)	13(65)
C07AB02 Metoprolol	1	20	9(45)	11(55)
**Class Total**	**8**	**204(100)**	**142(69.6)**	**62(30.4)**
D01BA02 Terbinafine	1	28	6(21)	22(79)
**Class Total**	**1**	**28(100)**	**6(21)**	**22(79)**
G04CA02 Tamsulosin	1	30	29(97)	1(3)
G04BD10 Darifenacin	1	28	23(82)	5(18)
G03DB01 Dydrogesterone	1	20	8(40)	12(60)
**Class Total**	**3**	**78(100)**	**60(76.9)**	**18(23.1)**
J01CR02 Amoxicillin and beta-lactamase inhibitor	16	224	136(61)	88(39)
J01XE01 Nitrofurantoin	3	90	27(30)	63(70)
J05AH02 Oseltamivir	6	60	31(52)	29(48)
J01FA09 Clarithromycin	3	42	22(52)	20(48)
J01DC04 Cefaclor	1	20	12(60)	8(40)
J01DC10 Cefprozil	1	20	14(70)	6(30)
J01FA10 Azithromycin	1	3	2(67)	1(33)
**Class Total**	**31**	**459**	**244(53.2)**	**215(46.8)**
M01AE17 Dexketoprofen	20	460	200(43)	260(57)
M01AB05 Diclofenac	19	320	150(47)	170(53)
M03BB53 Chlorzoxazone, combinations excl psycholeptics	9	180	87(48)	93(52)
M01AE09 Flurbiprofen	4	105	80(76)	25(24)
M01AB08 Etodolac	3	52	18(35)	34(65)
M01AE01 Ibuprofen	3	42	24(57)	18(43)
M01AG01 Mefenamic Acid	2	40	18(45)	22(55)
M01BX Other antiinflammatory/antirheumatic	2	40	20(50)	20(50)
M03BX30 Pheniramidol	1	24	4(17)	20(83)
M01AE02 Naproxen	2	20	10(50)	10(50)
M01AE03 Ketoprofen	2	20	12(60)	8(40)
M03BX05 Thiocholchicoside	1	14	12(86)	2(14)
**Class Total**	**68**	**1317(100)**	**635(48.2)**	**682(50.8)**
N02BE01 Paracetamol	18	360	153(43)	207(58)
N02BE51 Paracetamol, combinations excl psycholeptics	4	120	52(43)	68(57)
N07CA01 Betahistine	2	120	86(72)	34(28)
N02BA01 Acetylsalicylic Acid	4	110	37(34)	73(66)
N02BB02 Metamizole Sodium	4	70	24(34)	46(66)
N02CC07 Frovatriptan	1	6	5(83)	1(17)
**Class Total**	**33**	**786(100)**	**357(45.4)**	**429(54.6)**
P01AB01 Metronidazole	6	120	53(44)	67(56)
P01AB03 Ornidazole	1	10	5(50)	5(50)
**Class Total**	**7**	**130(100)**	**58(44.6)**	**72(55,4)**
R01BA52 Pseudoephedrine, combinations	23	636	292(46)	344(54)
R05X Other cold preparations	23	558	298(53)	260(47)
R05CB10 Combinations (Acetylcysteine, Vit C)	7	210	88(42)	122(58)
R05CB01 Acetylcysteine	6	140	89(64)	51(36)
R06AE07 Cetirizine	4	80	42(53)	38(48)
R06AX27 Desloratadine	3	60	9(15)	51(85)
R06AX29 Bilastine	3	60	16(27)	44(73)
R03DC53 Montelukast, combinations	1	30	27(90)	3(10)
R05DB13 Butamirate	1	20	17(85)	3(15)
R06AB05 Pheniramine	1	20	17(85)	3(15)
R06AX28 Rupatadine	1	20	10(50)	10(50)
**Class Total**	**73**	**1834**	**905(49.3)**	**929(50.7)**
**Total**	**265**	**6189**	**3132(50.6)**	**3057(49.4)**

Number of pharmaceuticals: Number of boxes of prescribed pharmaceuticals leftover after treatment, Number of Dosage Forms: Number of dosage forms in a box of pharmaceuticals, Used n(%): Used for treatment, Unused n(%): Leftover after treatment

## Discussion

Many studies have been conducted on unused medicines and their disposal practices. According to the findings of these studies, there is a serious public health problem and lack of policy. This study presents the current situation in Türkiye. In our study, there was a statistical difference between the number of unused drugs and some sociodemographic characteristics such as marriage, education, economic status etc. In the literature search, studies researching socio-demographic characteristics and the number of pharmaceuticals are very limited. A previous study found that medication disposal practice was found to be associated with some sociodemographic variables such as gender, age, marital status, and residence [16]. In another study, it was reported that parameters such as gender, education level, and place of residence were associated with drug keeping at home [17]. Therefore, it is possible to say that some sociodemographic characteristics are effective on the dependent variables.

According to the findings obtained from the participants’ practices on unused pharmaceuticals, it was found that pharmaceuticals were generally stored in the kitchen and refrigerator, and most of the pharmaceuticals were prescribed by primary health care facilities and family physicians. Addition, it was found that the most important reason for having unused pharmaceuticals in their households was reuse (for treatment), 44.6% kept them in household until the expiration date, 72.1% checked the expiration date, 56.7% read the package insert, and 41.3% purchased pharmaceuticals without a prescription. The findings that expired pharmaceuticals were thrown away, that improper disposal of pharmaceuticals polluted the environment and that the awareness on pharmaceuticals should be raised by the Ministry of Health came to the forefront. In a study reported by Manocha et al. (2020), it was reported that a significant proportion of participants had unused pharmaceuticals in their households and threw away expired pharmaceuticals [18]. In a study conducted by Althagafi et al. (2022), it was shown that about half of the participants were found pharmaceuticals in their households and that these pharmaceuticals were mainly stored in the refrigerator [4]. In another study researching the storage conditions of pharmaceuticals, it was shown that pharmaceuticals were stored in many different places, but especially in the kitchen [19]. In a study conducted by Gidey et al. (2020), it was reported that improper disposal of pharmaceuticals might be harmful to the environment and 77.4% disposed of pharmaceuticals by throwing them in household garbage [20]. In another study on medication disposal, it was reported that participants mostly disposed of medicines by throwing them in household garbage [21]. The dominant view was that unused pharmaceuticals were generally accumulated in households, expired pharmaceuticals were thrown in household garbage, pharmaceuticals were stored in the kitchen, and improper disposal harmed the environment in literature. When we compare the findings in the literature with our study findings, it is possible to say that the findings are similar. We think that the variation in the findings of behaviors towards unused medicines (storage, disposal, etc.) may be related to the type of study, number of participants, participant characteristics, culture, region and development level of the country.

According to the findings of our study, there were unused drugs in almost all ATC classes. In addition, the most prominent drugs in the quantity of leftover drugs were analgesics (paracetamol, etc.), cold medicines, NSAIDs (dexketoprofen, etc.), antibiotics (amoxicillin, etc.) and proton pump inhibitors (lansoprazole, etc.). According to various studies conducted based on the classification of the mechanism of action, drugs such as analgesics and pain relievers [22], antipyretics, analgesics, antispasmodics, antibiotics, antacids, and vitamins [23], antibiotics and pain-spasm relievers [24], antibiotics and non-steroidal anti-inflammatory drugs (NSAIDs) [12], antibiotics and analgesics [2], NSAIDs, antibiotics, and vitamins [13], were reported. In studies conducted in Mexico and Saudi Arabia, which are located in two different geographies, NSAIDs came to the forefront [25, 26]. It is seen that the findings of studies based on the mechanism of action of unused pharmaceuticals in the literature are similar to each other. In our study, ATC classification was performed and it can be said that the Turkish context also supports the existing literature.

According to the findings of our study, 50.6% of the total number of dosage forms of pharmaceuticals were used but 49.4% were unused. Patients should follow the treatment procedure prescribed by the physician. However, in some cases, medication may be stopped early by the patient. Some studies have reported that the most important reason for having unused or expired pharmaceuticals in household is recovery [1, 27]. Other reported reasons were a change in treatment by the doctor and feeling well [28]. In our study, we showed that approximately half of the medicines were not used. The presence of unused medicines in households might be related to self-medication. In our study the prevalence of self-medication was 94.1%. Analgesics, common cold and muscle relaxants of the pharmaceuticals were found to be the most commonly used pharmaceuticals without consulting a physician. This finding was reported by Kumar et al. (2013) as 78.6% [29], by Niromand et al. (2020) as 72% [30], by Abdi et al. (2018) as 89.6% [31], and by Bahzadifar et al. (2020) as 70.1%. In the same study, it was reported as 97.2% in medical students and 44.7% in non-medical students [32]. According to the findings of a local study, the prevalence of self-medication was 64.3%, and it was found that analgesics, antibiotics and cold pharmaceuticals were mostly used in this way [33]. In another study, analgesics, antibiotics and antacid pharmaceuticals came to the forefront [34]. When we compare the findings of previous studies with our finding, it is possible to say that a finding similar to the literature has emerged when the number and quantity of unused pharmaceuticals at home are taken into consideration and that the prevalence related to self-medication is one of the highest findings in the literature.

## Conclusion

According to findings, there is a significant quantity and number of unused pharmaceuticals with various active ingredients in households. It was observed that the unused pharmaceuticals were used without consulting a physician, were not disposed of properly, and some of them had expired. Considering the conditions associated with pharmaceuticals, this situation points to a significant public health problem for both people and environment. The public urgently needs to be informed about the use of pharmaceuticals. The accumulation resulting from any discontinuation of pharmaceutical therapy needs to be monitored and updated public health policies and legislation are required. In addition, a “drug take-back system” for unused pharmaceuticals may be useful in solving this problem.

### Limits of the study

This study has some limitations. The limitations of this study included the fact that a significant portion of the study data was collected by online method, that it was based on the statements and practices of the participants, and that some pharmaceuticals might not be reported by the participants considering that they were related to privacy and research data were collected from a single center.

## Data Availability

The datasets used and/or analysed during the current study available from the corresponding author on reasonable request.
